# Drought and heat stress mediated activation of lipid signaling in plants: a critical review

**DOI:** 10.3389/fpls.2023.1216835

**Published:** 2023-08-10

**Authors:** Parul Sharma, Nita Lakra, Alisha Goyal, Yogesh K. Ahlawat, Abbu Zaid, Kadambot H. M. Siddique

**Affiliations:** ^1^ Department of Botany and Plant Physiology, Chaudhary Charan Singh Haryana Agricultural University, Hisar, Haryana, India; ^2^ Department of Molecular Biology, Biotechnology and Bioinformatics, Chaudhary Charan Singh (CCS) Haryana Agricultural University, Hisar, India; ^3^ Division of Crop Improvement, Indian Council of Agricultural Research (ICAR)—Central Soil Salinity Research Institute, Karnal, India; ^4^ Department of Biological Sciences, Michigan Technological University, Houghton, MI, United States; ^5^ Horticultural Sciences Department, University of Florida, Gainesville, FL, United States; ^6^ Plant Physiology and Biochemistry Section, Department of Botany, Aligarh Muslim University, Aligarh, India; ^7^ Department of Botany, Government Gandhi Memorial (GGM) Science College, Cluster University Jammu, Jammu, India; ^8^ The UWA Institute of Agriculture, The University of Western Australia, Perth, WA, Australia

**Keywords:** lipid signaling, abiotic stress, lipid remodeling, phosphatidic acid, inositol phospholipids, stress tolerance

## Abstract

Lipids are a principal component of plasma membrane, acting as a protective barrier between the cell and its surroundings. Abiotic stresses such as drought and temperature induce various lipid-dependent signaling responses, and the membrane lipids respond differently to environmental challenges. Recent studies have revealed that lipids serve as signal mediators forreducing stress responses in plant cells and activating defense systems. Signaling lipids, such as phosphatidic acid, phosphoinositides, sphingolipids, lysophospholipids, oxylipins, and N-acylethanolamines, are generated in response to stress. Membrane lipids are essential for maintaining the lamellar stack of chloroplasts and stabilizing chloroplast membranes under stress. However, the effects of lipid signaling targets in plants are not fully understood. This review focuses on the synthesis of various signaling lipids and their roles in abiotic stress tolerance responses, providing an essential perspective for further investigation into the interactions between plant lipids and abiotic stress.

## 1 Introduction

The world’s growing population, increased per capita caloric intake, and growing need for renewable resources from plants have increased the demand for agricultural products (Singer et al., 2020). However, abiotic stressors, such as heat, cold, and drought, have a detrimental effecton crop yields, and their frequency and severity are increasing due to climate change (Myers et al., 2017; Zaid et al., 2022). As sessile organisms, plants are subjected to various biotic and abiotic stresses, typically sensed through the plasma membrane that contains signaling lipids. The modification of enzymes such as phosphatases, phospholipases, or lipid kinases generates stress signals (Testerink and Munnik, 2011), which are translated into biological reactions. In recent years, lipids have gained significant attention as an essential element of biological membranes in all plant tissues, particularly in response to stresses. Membrane lipid modification is an efficient adaptation method for plants to defend against various abiotic stimuli, including drought, salt, cold, heat, nutritional deficiencies, and intense psychological pressures. The ability of plants to adapt to various environmental challenges depends on their ability to respond to various abiotic stressors by altering membrane lipids (Liu et al., 2019). Increasing evidence suggests that lipids play a role in the alleviation of stress responses in plant cells and the activation of defensive systems as signal mediators (Gaude et al., 2008; Nakamura et al., 2009; Moellering et al., 2010; Gasulla et al., 2013; Okazaki and Saito, 2014). Lipids can be categorized into eight major classes based on their hydrophobic and hydrophilic components: fatty acids, glycolipids, glycerolipids, glycerophospholipids, sphingolipids, sterol lipids, prenol lipids, saccharolipids and polyketides (Fahy et al., 2005). Signaling lipids, including lysophospholipids, fatty acids, phosphatidic acid (PA), diacylglycerol, oxylipin, sphingolipid, and N-acylethanolamine (Wang and Chapman, 2013), are generally found in minute concentrations in tissues. Diacylglycerol pyrophosphate (DGPP) is generated by the process of phosphorylating PA and is typically not present in non-stimulated cells, but its concentration rapidly rises within minutes upon exposure to various stimuli such as osmotic stress which suggests that DGPP plays a significant role in signaling pathways related to stress responses (Van Schooten et al., 2006).Esterase and other lipid hydrolytic enzymes, such as phospholipases, are crucial in signaling lipid formation from pre-existing membrane lipids or membrane lipid biosynthetic intermediates (Wang et al., 2006; Munnik and Testerink, 2009). Triacylglycerol (TAG), which has a glycerol backbone and three esterified fatty acids, is primarily kept in plants as a high-energy storage substance in lipid droplets in seeds or fruits (Xu and Shanklin, 2016). TAG does not typically accumulate insignificant amounts in vegetative tissues under non-limiting growth conditions, but various stress events, such as dehydration and extreme heat or cold, can stimulate its formation, particularly in leaves (Lee et al., 2019).

Chloroplasts are typically the first abiotic damage sites observed in plant ultrastructure. Chloroplast deterioration decreases the net photosynthetic rate and plant growth (Janik et al., 2013). Temperature and drought stress can permanently alter chloroplast structure by reducing their size and aspect ratio and changing the membrane phase (Varone et al., 2012), compromising their integrity and fluidity and rendering the chloroplasts inactive (Upchurch, 2008; Han et al., 2010). Photosynthetic membrane lipids play a significant role in preserving chloroplast structural integrity by maintaining the grana lamellar structure stack, stabilizing membranes, and facilitating the dense packing of proteins in the membrane (Garab et al., 2000; Gaude et al., 2007; Wang et al., 2014). Membrane lipids plays a significant role under stresses by preserving the chloroplast’s lamellar stack, stabilizing the chloroplast membranes (Shimojima and Ohta, 2011), making it easier to pack the membrane’s proteins, and controlling membrane fluidity by adjusting the degree of fatty acid desaturation (Dakhma et al., 1995; Zhang et al., 2005; Sui et al., 2010; Wang et al., 2010; Barnes et al., 2016). The thylakoid lipid bilayer mainly comprises four distinct lipids: monogalactosyldiacylglycerol (MGDG), digalactosyldiacylglycerol (DGDG), sulfoquinovosyldiacylglycerol (SQDG), and phosphatidylglycerol (PG) (Moellering and Benning, 2011). MGDG and DGDG are uncharged galactolipids, forming the main body of thylakoid membrane lipids, and provide a lipid bilayer matrix as the main component for photosynthetic complexes (Shimojima et al., 2015).

In response to stresses like cold, dehydration, and nutrient loss, membrane lipid modifications take place, helping maintain membrane characteristics impacting lipid dynamics, membrane integrity, and membrane-bound protein activities (Samuels et al., 2008). Lipids also serve as intermediates in signal transduction pathways, and their role as signaling molecules is gaining attention. This review emphasizes the function of six prominent signaling lipids—phosphatidic acid (PA), phosphoinositides (PI), sphingolipids, lysophospholipids, oxylipins, and N-acetylethanolamines—and their roles in abiotic stress responses.

## 2 Signaling lipids biosynthesis in plants

### 2.1 Phosphatidic acid

**Figure 1:**
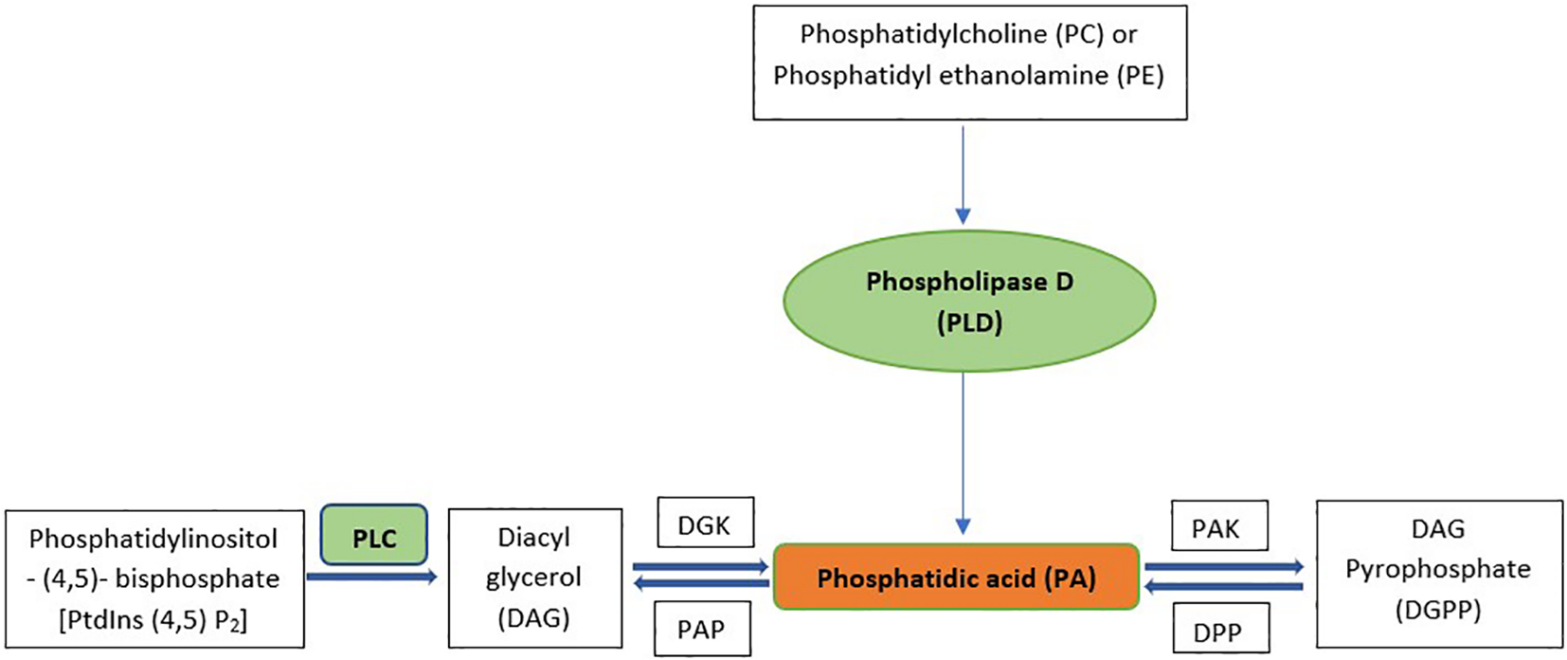
Synthesis Pathways for signaling PA. PLC, Phospholipase D; DGK, Diacyl glycerol kinase; PAK, Phosphatidic acid kinase; PAP, Phosphatidic acid phosphatase; DPP, DGPP phosphatase.

Phosphatidic acid (PA), a diacyl glycerophospholipid, functions as a cellular signaling molecule and acts as a precursor for the synthesis of complex lipids (Wang et al., 2016). PA-based structural phospholipids and glycolipids are synthesized in the endoplasmic reticulum, plastids, and mitochondria, but PA as a signaling molecule is mostly derived from various phospholipase pathways in the plasma membrane (Testerink and Munnik, 2011).Two different phospholipase pathways synthesize phosphatidic acid, which has a significant role in cell signaling (Arisz et al., 2009; Bargmann et al., 2009a; Hong et al., 2009; Li et al., 2009). The first pathway involves the direct production of PA by the enzyme phospholipase D (PLD), which hydrolyzes phosphatidylcholine (PC) and phosphatidylethanolamine (PE) structural lipids to produce PA and the remaining headgroup (Pappan et al., 1998; Hong et al., 2008). The second pathway generates PA through the subsequent actions of the enzymes phospholipase C (PLC) and diacylglycerol kinase (DGK). PLC converts inositol-1,4,5-trisphosphate (Ins(1,4,5)P_3_) and DAG from phosphatidylinositol-4,5-bisphosphate (PtdIns(4,5)P_2_). Ins(1,4,5)P_3_ diffuses into the cytosol, while DAG remains in the membrane and is promptly phosphorylated to PA by DGK. PA phosphatase(PAP) then converts PA back into DAG and Ins(1,4,5)P_3_. PA kinase (PAK) converts PA into DAG pyrophosphate (DGPP), which diminishes the signal. DGPP phosphatase(DPP) is the enzyme that converts DGPP back into PA (Munnik et al., 1996; Meijer and Munnik, 2003). **Figure 1** shows the biosynthetic pathway of PA.

### 2.2 Phosphoinositides

Phosphoinositides (PI) are a group of lipids that serve as cellular signaling molecules and are produced from phosphatidylinositol (PtdIns) by lipid kinases and phosphatases. They also may act as precursors to second messengers. Seven different isoforms of PI are produced based on the location of phosphates on the inositol ring, including three monophosphates, three bisphosphates, and one trisphosphate- PtdIns3P, PtdIns4P, PtdIns5P, PtdIns(3,4)P2, PtdIns(3,5)P2, PtdIns(4,5)P2 and PI(3,4,5)P3 (Meijer and Munnik, 2003). In plants, PIPs constitute significantly less than 1%, with PI(4)P the most prevalent, followed by PI(4,5)P2 (Balla, 2013; Hou et al., 2016).

The discovery of the PIP second messenger system in plants dates back to 1985 when PI(4)P and PI(4,5)P2, collectively known as the PIP second messenger system, were identified for the first time in plants using carrot suspension cultures and thin-layer chromatography of 3H radiolabelled phospholipids (Boss and Massel, 1985). Further studies using high-performance liquid chromatography of radio-labelled samples led to the isolation of PI(3)P from *Spirodelaolyrhiza* L., raising the possibility of the PI3K kinase signaling pathway in plants. In 1997, PI(3,5)P2 was discovered in carrot suspension cells under osmotic stress with NaCl (Dove et al., 1997), and in 2001, PI(5)P was identified in *Arabidopsis thaliana* plants under water stress. However, certain stress conditions may be required for the cells to produce other PIs, such as PI(3,4)P2 and PI(3,4,5)P3, which have not yet been discovered in plants. Nonetheless, some plant-specific proteins may be able to bind or synthesize these PIs under certain conditions; for example,AtPTEN1 binds to PI(3)P and PA *in vivo* and PI(3,4,5)P3 *in vitro*, while AtPIP5K1 kinase synthesizes PI(3,4)P2 from PI(3)P and PI(3,4,5)P3 *in vitro* (Zhang et al., 2011).

**Figure 2:**
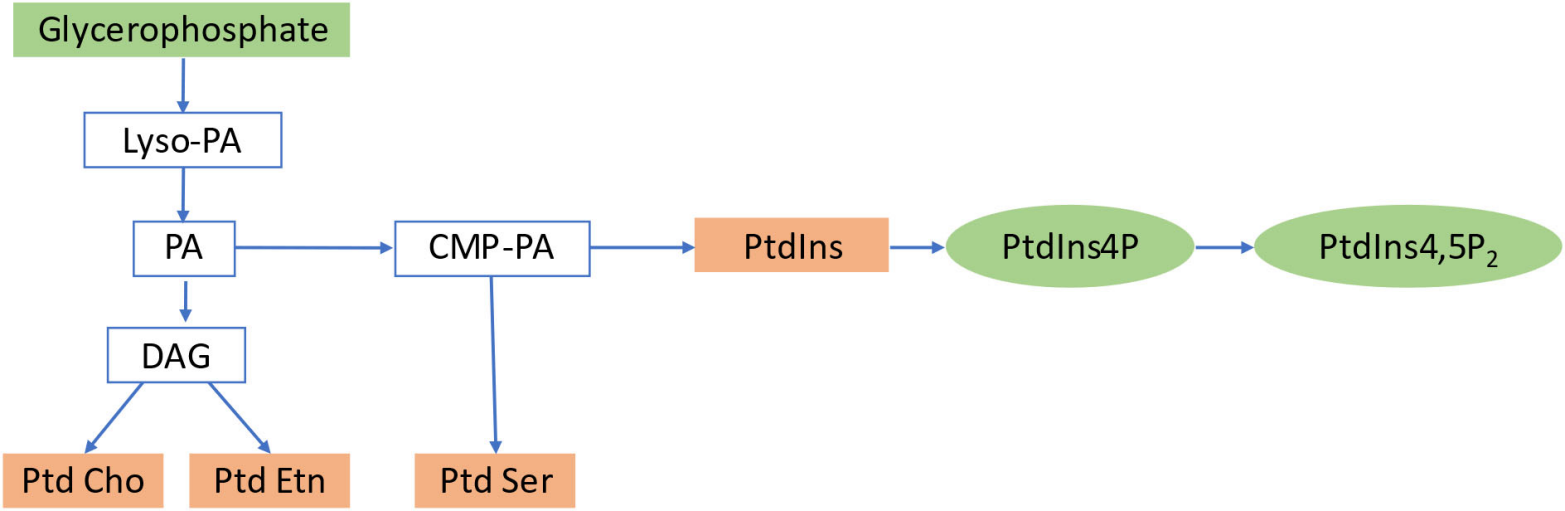
Biosynthetic pathway of Phosphoinositides. PA, Phosphatidic Acid; DAG, Diacylglycerol; PtdIns, Phosphoinositides; PtdCho, Phosphatidylcholine; PtdEtn, Phosphatidylethanolamine; PtdSer, Phosphatidylserine; PtdIns4P, Phosphatidylinositol 4 phosphate; PtdIns4,5P2, Phosphatidylinositol4,5 Bisphosphate.

Inositol phosphates (IPs) and diacylglycerol (DAG) are produced from phosphoinositides by PI-specific PLCs. While DAG is extensively studied as a signaling lipid in mammals, its function in plants remains unclear. DAG kinases in plants can phosphorylate DAG further, resulting in the production of PA (Arisz et al., 2013). **Figure 2** summarizes the formation of PIs by sequential acylation of glycerophosphate and lyso-phosphatidic acid. Inositol 1,4,5-triphosphate (IP3), the most prevalent IP in plant cells, regulates various cellular processes, including plant development and stress responses (Franklin-Tong et al., 1996; DeWald et al., 2001), and may regulate cellular reactions by inducing Ca^2+^ production in the cytoplasm of guard cells to increase Ca^2+^ levels and closestomata (Sanders et al., 1999). Plants use IP3 as a precursor to produce inositol hexakisphosphate (IP6), which could be the true signaling molecule in plants (Tsui and York, 2010; Williams et al., 2015). IP6 has a critical role in guard cell responses, leading to stomatal closure (Lemtiri-Chlieh et al., 2003). IP5 and IP6 have also been identified as structural co-factors of the auxin receptor–transport inhibitor response 1 (TIR1) and jasmonic acid receptor-coronatine insensitive 1 (COI1), respectively (Sheard et al., 2010), connecting phytohormone-controlled pathways with PI signaling. PIs are involved in various cellular functions, including membrane trafficking, cytoskeleton organization, polar tip growth, and stress responses (Ischebeck et al., 2010). The *Arabidopsis* genome encodes 20 PtdIns kinases that generate different PtdIns isoforms. In addition to phosphorylating PtdIns4P, PI4P 5-kinases also catalyze the unspecific process of phosphorylating PtdIns3P, resulting in PtdIns(3,5). The synthesis of PtdIns(4,5) P2 is likely catalyzed by PI4P 5-kinase using PtdIns4P as a substrate, whereas PtdIns5P is likely generated by the dephosphorylation of PtdIns bisphosphate. However, no gene encoding PI 5-kinase or PI5P 4-kinase has been found in plants (Heilmann and Heilmann, 2015).

### 2.3 Sphingolipids

**Figure 3:**
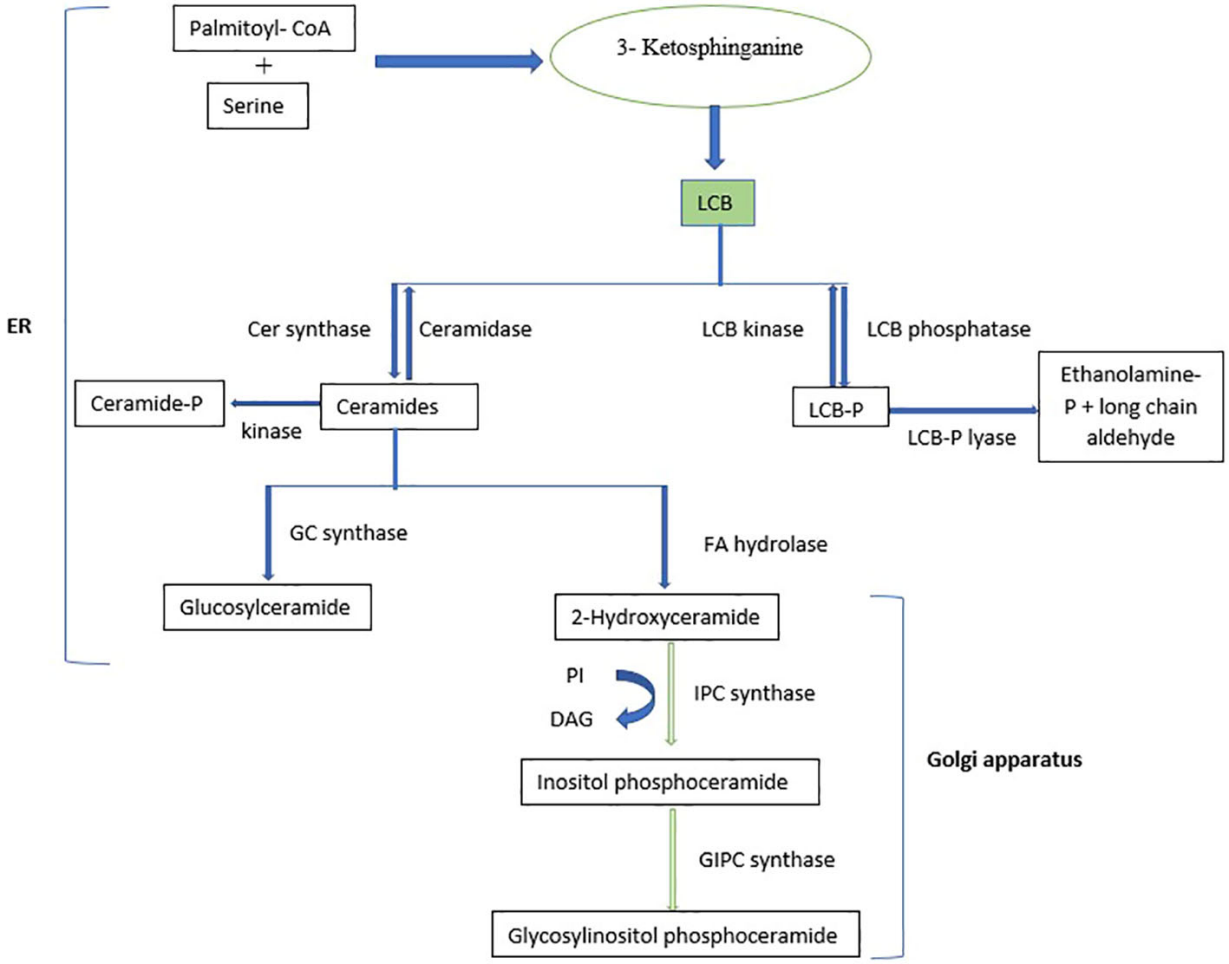
Biosynthetic pathway of sphingolipids. LCB, Long-chain base; PI, Phosphatidylinositol; DAG, Diacylglycerol.

Sphingolipids are a highly diverse group of compounds involved in numerous cellular processes (Pata et al., 2010). They constitute a significant proportion of the lipid content in higher plants and are abundant in endosomes and tonoplasts, making up to 40% of the lipids in the plasma membrane (Moreau et al., 1998; Cacas et al., 2016). Sphingolipids are structurally diverse, comprising asphingoid long-chain base (LCB) backbone amide joined to an N-acylated fatty acid (FA) attached to a polar head group (Gault et al., 2010; Pata et al., 2010). Sphingolipid metabolism occurs mainly in the endoplasmic reticulum (ER) and the golgi apparatus, where they are synthesized, transferred, sorted, transported, and eventually localized to membranes (Luttgeharm et al., 2016). In plants and yeast, dihydrosphingosine and phytosphingosine are the main LCBs (Hannun and Obeid, 2008). Plant sphingolipids mainly comprise glucosylceramides (GlcCers) or glycosylated inositolphosphoryl ceramides (GIPCs) (Markham et al., 2006). **Figure 3** shows the biosynthesis pathway of sphingolipids, which involves the condensation of serine and palmitoyl-CoA catalyzed by serine palmitoyl transferasein the ER to produce various LCBs, typically with 18 carbons (Chen et al., 2006), followed by the reduction of 3-ketosphinganine to sphinganine (d18:0) by 3-ketosphinganine reductase (Beeler et al., 1998). Sphingosine N-acyltransferase then joins the LCBs to a fatty acid and further modifies them to create ceramides, which serve as the building blocks for more complex sphingolipids (Spassieva et al., 2002; Merrill, 2011). Ceramides are synthesized in the ER and transported to the Golgi, where they are modified by the attachment of various polar head groups to form GlcCers, inositolphosphoryl ceramides, and GIPCs (Berkey et al., 2012).

Sphingolipids are highly concentrated in the cytoplasm and vacuolar membrane, creating membrane microdomains called lipid rafts, crucial for protein trafficking to the plasma membrane and other cell surface activities (Simons and Toomre, 2000; Pike, 2006). Sphingolipids must be transported from the ER to the Golgi network and then to plasma and vacuolar membranes to produce and translocate complex GIPCs. However, sphingolipid transport in plant cells is not well understood. Studies suggest that accelerated cell death protein 11 and glycolipid transfer protein 1 might be involved in sphingolipid transport in plants (Simanshu et al., 2014), but further research is needed to confirm this. Several studies have revealed information on the role of sphingolipids in plant development and in response to diverse abiotic and biotic stresses (Markham et al., 2013; Luttgeharm et al., 2016; Ali et al., 2018; Huby et al., 2020; Mamode Cassim et al., 2020; Liu et al., 2021).

Sphingolipids play a key role in programmed cell death (PCD) during immune response or plant development. The accumulation of LCBs stimulates CPK3, a calcium-dependent kinase, which then phosphorylates its 14-3-3 protein binding partners and triggers PCD (Lachaud et al., 2013). Moreover, LCB-Ps are involved in ABA, cold, and drought stress responses (Worrall et al., 2008; Guillas et al., 2013). While the role of sphingolipids in animals is well understood, the sphingolipid signaling system in plants is still largely unknown. In plants, complex sphingolipids such as GlcCers and GIPCs play a structural role similar to sphingomyelin in mammals and are important structural elements of cell membranes, but their role in signaling is not yet known. Researchers have questioned whether plants have an enzymatic breakdown pathway for GIPCs, similar to the one found in mammals (Worrall et al., 2003). The answer to this question could provide a fresh perspective on how sphingolipid signaling functions in plants.

### 2.4 Lysophospholipids

**Figure 4:**
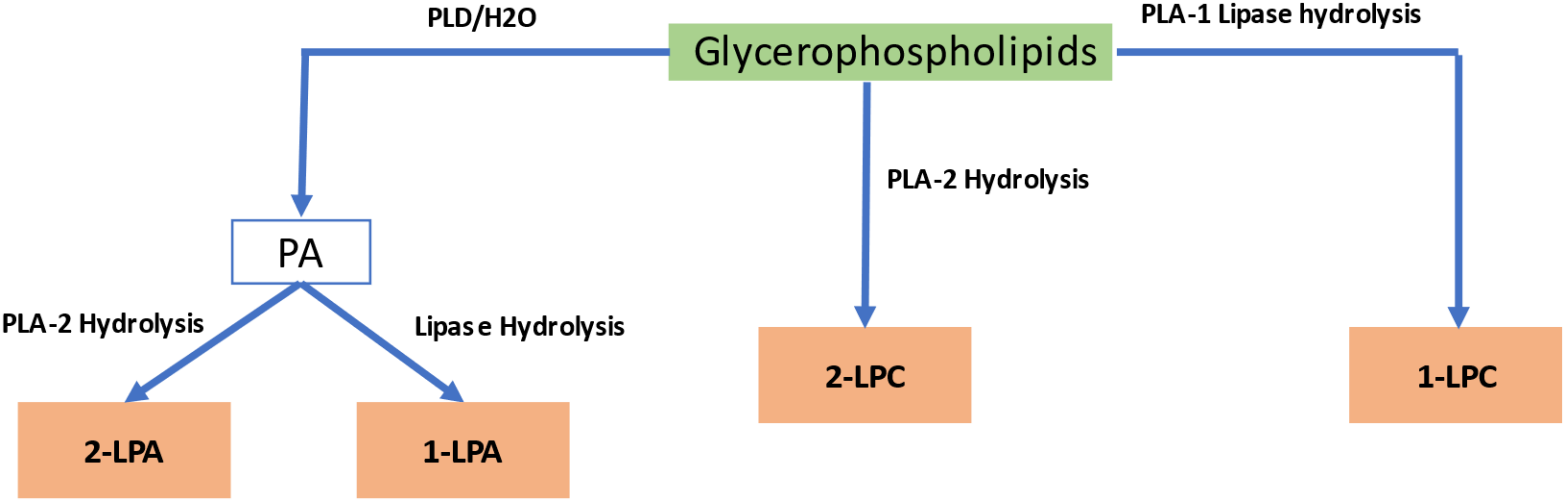
Biosynthetic pathway of lysophospholipids. PA, Phosphatidic Acid; LPA, lysophosphatidic acid; LPC, lysophosphatidylcholine.

Lysophospholipids are generated from glycerophospholipids M.via phospholipase A catalysis, leading to a lipid containing only a single acyl chain, such as lysophosphatidylcholine (LPC), lysophosphatidic acid (LPA), and sphingosylphosphorylcholine. **Figure 4** summarizes the process of enzymatic production of LPs by phospholipase A1 (PLA1), phospholipase A2 (PLA2), and lipases. When natural phosphatidic acid (PA) or phosphatidylcholine (PC) undergo a hydrolysis reaction with phospholipase A2 (PLA2), the resulting products are 2-lysophosphatidic acid (2-LPA) and 2-lysophosphatidylcholine (2-LPC) (Kim and Kim, 1998).They are abundant in the membrane’s lipid bilayer, with glycerol and sphingosine as their main components, and have various details that determine their cell receptor selection, including the position of the acyl chain on the glycerol moiety, the length, degree, and saturation of the fatty acyl chain, and the phosphate head group. Lysophospholipids accumulate in plants in response to freezing, wounding, or pathogen infection (Wi et al., 2014).

Lysophospholipids play a crucial role in pollen growth, stomatal opening, and reactions to hypoxia and salt stress (Wang et al., 2019). Analyzing the regulatory mode and function of phospholipase A (PLA) will help understand the signaling cascades involving lysophospholipids as PLA1 and PLA2,the essential enzymes for producing lysophospholipids. G-proteins have been shown to regulate PLA2 activities in plants (Heinze et al., 2013). However, LPCs can cause a cytoplasmic pH change by activating tonoplast H^+^/Na^+^ antiporter activity and H^+^-transporting ATPase of the plasma membrane, which affect auxin responses (Viehweger et al., 2002). According to one study, plants may have evolved H^+^-ATPases as lysophospholipid sensors (Wielandt et al., 2015), suggesting that lysophospholipid signaling receptors are distinct in plants and animals. Moreover, PLA2 expression is down regulated during heat and drought stress and upregulated during salt, osmotic, and cold stress (Hruz et al., 2008). Despite this, the function and regulatory mechanism of lysophospholipids are still poorly understood, in contrast to the extensively studied PA signaling molecule.

### 2.5 Oxylipins

**Figure 5:**
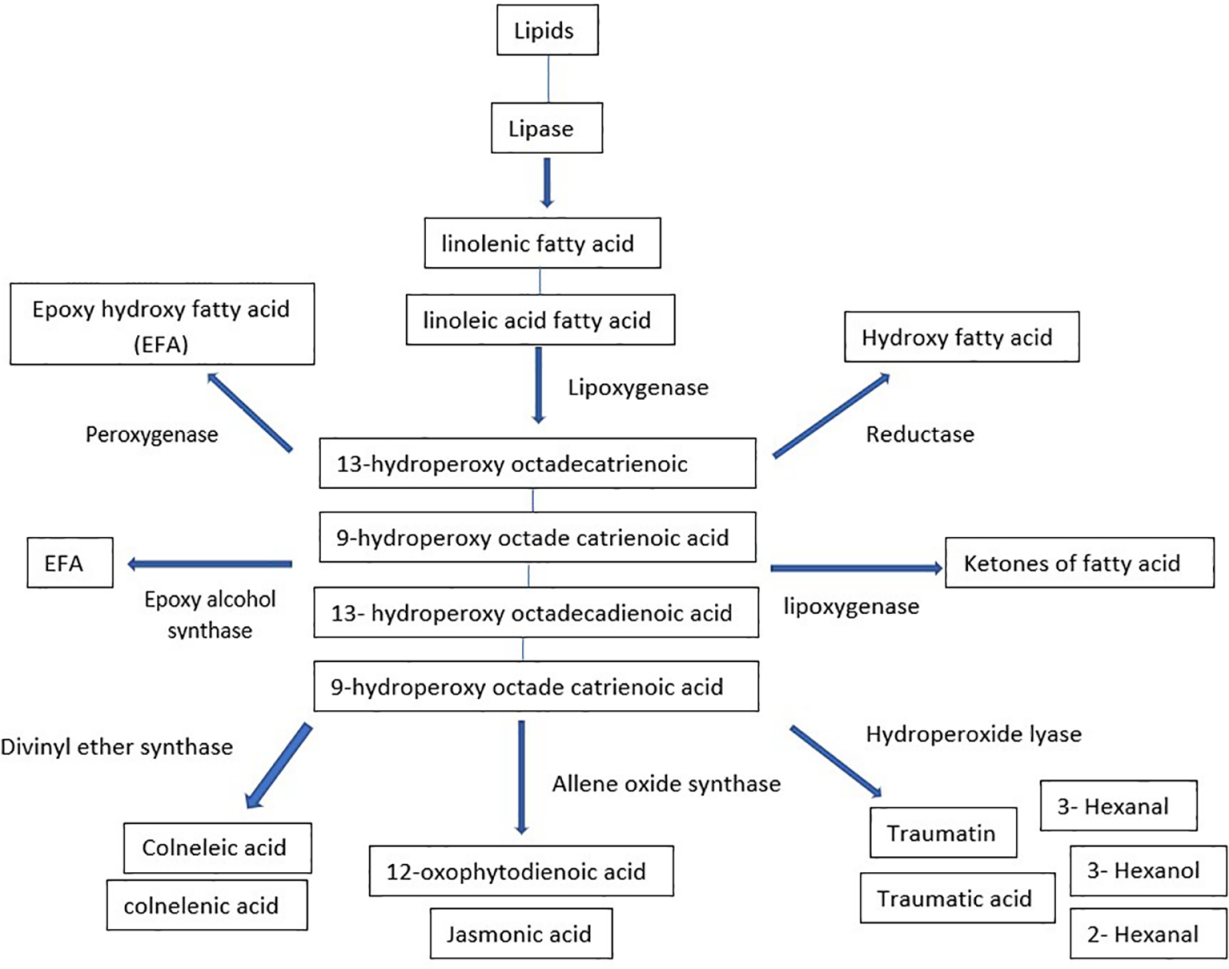
Biosynthetic pathway of oxylipins.

Oxylipins are a broad category of compounds that perform various functions in plant growth, development, and interactions with biotic and abiotic stresses. The most extensively studied subgroup of plant oxylipins is jasmonates (JAs), commonly known as defense phytohormones. While the biosynthetic pathway is very straight forward, the metabolites produced are structurally diverse and biologically active. Oxylipins interact with various signaling pathways in plant cells, including auxin, gibberellin, ethylene, and ABA signaling pathways involved in regulating stress-induced gene expression and stress signal transduction (Yang et al., 2012). Oxylipins are comprised of complex oxidized fatty acids such as fatty acid hydroperoxides, divinyl ethers, and jasmonic acid. They can be synthesized enzymatically or spontaneously through auto-oxidation (Andreou et al., 2009; Mosblech et al., 2009). Lipoxygenases (LOXs) (e.g., jasmonates) or 18-carbon unsaturated fatty acids, such as linolenic acid or linoleic acid, are typically involved in oxylipin production in plants (Savchenko et al., 2014). The biosynthesis process can be divided into various classes using synthesized fatty acid hydroperoxides as substrates for various enzymes (**Figure 5**). Hydroperoxide lyase produces aldehydes, alcohols, and oxo-acids that play important roles in pathogen defense, and allene oxide synthase that catalyzes the process leading to JA signaling (Griffiths, 2015). Peroxygenases (POX) and divinyl ether synthases synthesize epoxy-hydoxy FA and divinyl ethers of fatty acids used in antimicrobial drugs. Epoxy alcohol synthases and LOXs are involved in synthesizing epoxy hydroxy fatty acids and keto fatty acids (Mosblech et al., 2009). Hydroxyl fatty acids can also be synthesized in the reduction of hydroperoxides by a reductase (Andreou et al., 2009).Oxylipin production may already have begun before a lipase releases fatty acids. Evidence suggests that lipoxygenase uses esterified lipid-bound fatty acids as substrates. In *Cannabis sativa*, there might exist a partially shared biosynthetic pathway in oxylipins and cannabinoids production. In the plant, a specific examination of trichomes’ gene expression revealed that the genes responsible for producing oxylipins, namely LIPOXYGENASE (LOX) and HYDROPEROXIDE LYASE (HPL), were found to be co-expressed with genes already known to be involved in the production of cannabinoids (Stout et al., 2012; Livingston et al., 2020). This observation has led to the hypothesis that the oxylipin pathway provides the necessary substrate for the production of cannabinoids (Borrego et al., 2023).

Another subclass of oxylipins is phytoprostanes, produced through a biochemical process in volving free radicals and non-enzymatic gtransformation of α-linolenic acid (Griffiths, 2015). During photosynthesis, the reactive oxygen species (ROS) produced in chloroplasts promote fatty acid oxidation in green leaves, with photosynthetic tissue containing ten times more phytoprostanes than roots. Phytoprostanes are likely formed from fatty acid residues in lipids, much like animal isoprostanes, and then released by lipases (Imbusch and Mueller, 2000). Phytoprostanescan regulate the expression of genes involved in detoxification processes and stimulate the production of secondary metabolites. A unique class of oxylipins known as reactive electrophile species includes 12-OPDA, certain phytoprostanes, fatty acid ketodienes and ketotrienes, 2(E)alkenals, and other unsaturated carbonyl compounds. These compounds have high chemical reactivity due to the electrophilicity of the carbonyl group’s double bond. ROS attack the nucleophilic areas of organic compounds, including glutathione, proteins, and nucleic acids, altering their characteristics and damaging cell structures. ROS also stimulate the production of genes involved in cell cycle regulation, xenobiotic, and excessive light defense responses (Farmer and Mueller, 2013).

### 2.6 N-acylethanolamines

**Figure 6:**
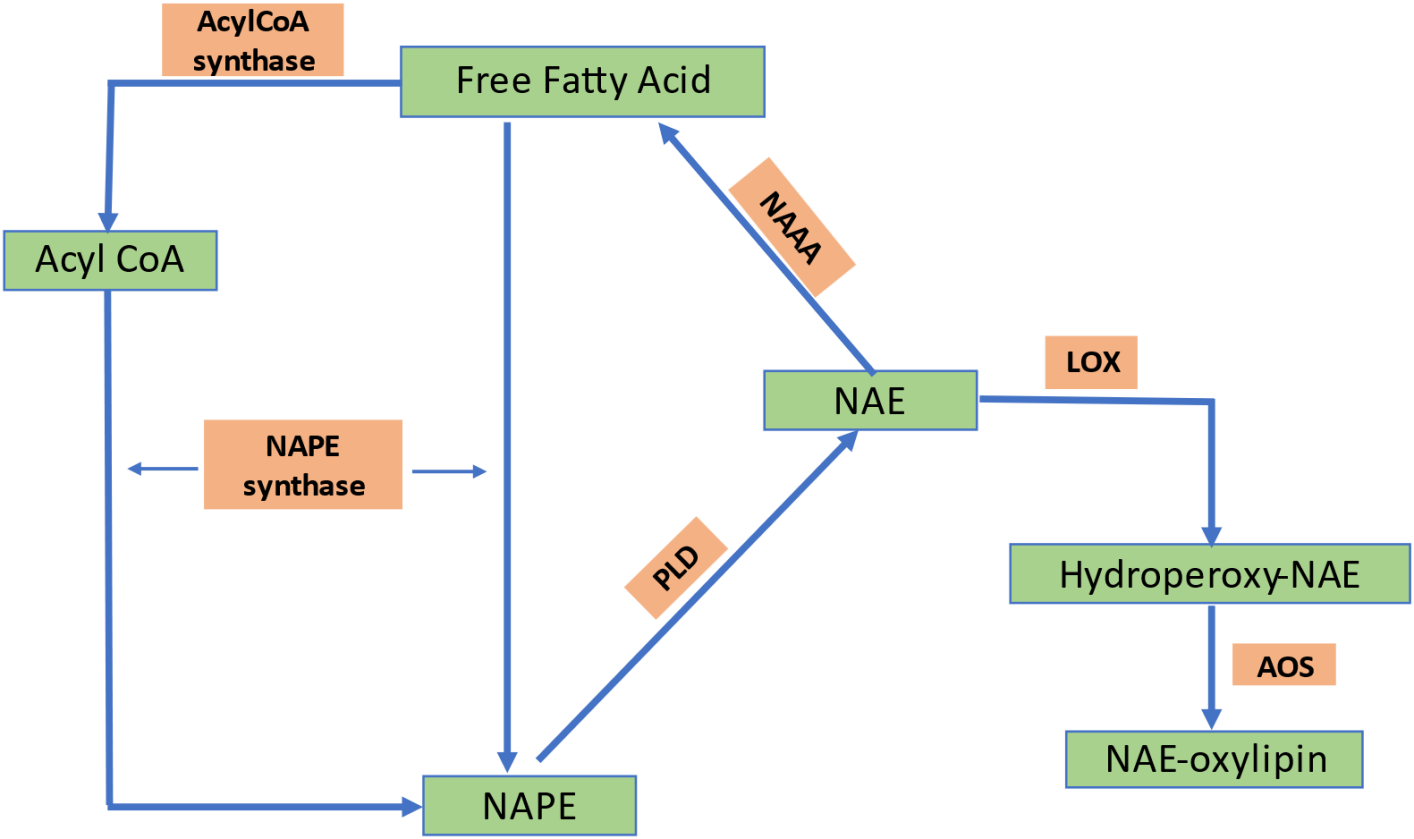
Biosynthetic pathway of NAE formation in plants. NAPE, N-acylphosphatidylethanolamine; PLD, phospholipase D; NAE, N-acylethanolamine; LOX, lipoxygenase; AOS, allele oxide synthase; PA, phosphatidic acid; NAAA, N-acylethanolamine-hydrolyzing acid amidase; FAAH, fatty acid amide hydrolase; PE, phosphatidylethanolamine.

N-acylethanolamines (NAEs) are a family of signaling lipids with a wide range of functional properties. They are categorized according to the number of carbons and saturation level in their acyl chains and comprise a fatty acid linked to ethanolamine by an amide bond. **Figure 6** shows the biosynthesis of NAEs (Arias-Gaguancela and Chapman, 2022).The most extensively researched NAE is N-arachidonylethanolamine, often known as anandamide, which functions as an endogenous ligand for cannabinoid (CB1 and CB2) receptors located on the plasma membrane in the brain and activates intracellular signaling cascades in mammalian neurons (Felder et al., 1993). Other more prevalent types of NAEs, such as ethanolamides of oleic, linoleic, linolenic, and palmitic acids, have also drawn attention as lipid mediators that primarily work without the assistance of G-protein-coupled cannabinoid receptors (Movahed et al., 2005). NAE concentrations vary depending on the stage of plant growth and development (Chapman, 2004).Among the different plant tissues examined, it was found that the content of NAE is highest in desiccated seeds of various plant species. However, the quantities are typically at low levels, measured in parts per million (ppm) (Chapman, 2004; Venables et al., 2005; Kilaru et al., 2007). According to radio-labeling research, plants use nacylphosphatidylethanolamines (NAPE) as a metabolic substrate of naphthalene (NAE) (Chapman et al., 1999). Animals can hydrolyze NAPE directly by a PLD to produce NAEs or indirectly through PLC- or PLA2/α/β-hydrolase-4 (ABHD4)-mediated pathways. PLC and PLA2/ABHD4 hydrolyze NAPE into phospho-NAE and lyso-NAPE, respectively. ABHD4 further hydrolyzes lyso-NAPE to produce glycerophospho-NAE (GP-NAE). Phosphatases and phosphodiesterasesthen convert GP-NAE and phospho-NAE to NAE, respectively. However, the biosynthesis pathway of NAE in plants is still unknown because several enzymes required for mammalian NAE synthesis pathways are absent inplants or have little in common with proteins found in animals (Blancaflor et al., 2014). Several NAE isoforms impact the regulation of plant development and pathogen defense (Wang et al., 2006; Kang et al., 2008). However, unlike animals, plants have little information about the perception and molecular targets of NAEs.

## 3 Lipid binding proteins

Plants had to develop mechanisms to detect changes in their environment and communicate these changes to different organs, and adjust accordingly (Thomas and Vince-Prue, 1996). These responses can occur within one cell or tissue or at a location distal from the detection of those environmental changes. The complexity of lipid metabolism becomes more apparent when considering that each membrane in a plant has a distinct composition of acyl and lipid. Additionally, all acyl-chains are produced in plastids and are assembled either in plastids or the endoplasmic reticulum, necessitating the transport of fatty acids and lipids. Since hydrophobic molecules cannot freely move in a watery cellular environment, multiple modes of transportation are employed such as diffusion or flip transfer within the same membrane system, vesicular trafficking, and transport processes facilitated by proteins. Lipid transfer proteins (LTPs) have a crucial function in aiding the transfer of phospholipids and galactolipids from one membrane to another (Kader, 1996). These proteins are alternatively referred to as nsLTPs (non-specific lipid-transfer proteins) or pLTPs (plant lipid transfer proteins). The peptides within this category possess a hydrophobic cavity resembling a tunnel that can accommodate various lipids (Edqvist et al., 2018). NsLTPs can be characterized as proteins that induce the relaxation of the cell wall (Nieuwland et al., 2005) by increasing its internal volume through turgor. Studies have demonstrated that annexins proteins possess the ability to interact with phospholipids and membranes. The expression of *Arabidopsis* annexin 1 is increased in response to both salt and water stress conditions. Plant Acyl CoA binding proteins (ACBPs) have the ability to bind long-chain acylCoA esters (Brown et al., 1998)., participate in acyl-CoA transport (Johnson et al., 2002), maintain the acyl-CoA pool (Yurchenko et al., 2014). Another way to move the long-distance signals is through plant phloem. The view of the phloem function has evolved from that of simple assimilate transport to a complex trafficking system for stress signals and developmental regulators. A recent analysis of phloem exudates of *Arabidopsis thaliana*, led to the identification of several glycerolipids within the phloem including PA, PC, PI, di- and triacylglycerols (Benning et al., 2012; Tetyuk et al., 2013). Similarly, lipids have been detected in canola phloem as well (Madey et al., 2002). **Table 1** summarizes various lipid binding proteins and their functions.

**Table 1:** Lipid binding proteins and their functions.

Lipid binding proteins	Functions		References	
Phosphatidic Acid Binding Proteins				
PDK1	Root hair development, defense against pathogens		Deák et al., 1999; Anthony et al., 2004; Anthony et al., 2006	
CDPK	Protein kinase		Farmer and Choi, 1999	
TGD2	Chloroplast structure		Awai et al., 2006	
PEPC	Phosphoenolpyruvate carboxylase		Testerink et al., 2004	
14-3-3 protein	Plasma membrane H^+^ATPase pathway		Testerink et al., 2004	
PID	PIN localization		Zegzouti et al., 2006	
AGD7	ER/Golgi trafficking		Min et al., 2007	
CTR1	Ethylene response		Testerink et al., 2007	
TPM1	Lipid metabolism		Jost et al., 2009	
MPK6	Abiotic and biotic stress signaling		Yu et al., 2010	
RbohD/F	ABA signaling		Ma et al., 2012	
PTEN2A	Lipid phosphatase activity		Pribat et al., 2012	
TGD4	Chloroplast structure		Wang et al., 2012; Wang et al., 2013	
WER	Root hair formation		Yao et al., 2013	
GAPC	Metabolism		Kim et al., 2013; McLoughlin et al., 2013	
RCN1	Auxin/BR signaling, root development		Gao et al., 2013; Wu et al., 2014	
PARP1	Lipid transport/Co-signal		Hyun et al., 2014	
Annexin	Lipid transport/Co-signal		Nakamura et al., 2014	
GDSL-lipase	Lipid release/Phloem entry		Barbaglia et al., 2016	
PLAFP 1	Lipid transport/Co-signal		Barbaglia et al., 2016	
PIG-P like protein	Receptor function		Barbaglia et al., 2016
LHY, CCA1	Circadian clock regulation		Kim et al., 2019
AHL4	Lipid mobilization/Seedling establishment		Cai et al., 2020
Phosphatidylinositol Binding Proteins				
LTP1	Cutin biosynthesis		Cameron et al., 2006	
ACBP2	Heavy metal stress tolerance		Gao et al., 2010	
P4M, PTB, PX	Biogenesis of vesicles, cell division, and defense response		Szumlanski and Nielsen, 2010; Munnik and Nielsen, 2011	
BATS, FYVE	Plant growth and development, membrane biogenesis and trafficking		Munnik and Nielsen, 2011; Gillaspy, 2013	
PH, PROPPIN	Function unknown, putative role in stomatal closure		Bak et al., 2013	
PCaP1 and PCaP2	Stomatal closure		Nagata et al., 2016
FAB1	Maintaining membrane trafficking		Hirano and Sato, 2019
Sphingolipid Binding Proteins				
ACD11	Sphingolipid transport		Brodersen et al., 2002	
GLTP1	Sphingolipid transport		West et al., 2008	
PDLP5	Sphingolipid translocation		Liu et al., 2020	
Lysophospholipid Binding Proteins				
LTP1	Cutin biosynthesis		Cameron et al., 2006	
LTP2	Suberin Biosynthesis		Edstam and Edqvist, 2014	
ACBP2	Heavy metal stress tolerance		Gao et al., 2010	

PDK1- phosphoinositide-dependent protein kinase 1, CDPK- Ca2+-dependent protein kinase, TGD- trigalactosyl diacylglycerol, PEPC- phosphoenolpyruvate carboxylase, PID- PINOID, AGD7- ARF gap domain, CTR1- constitutive triple response 1, TPM1- tropomyosin alpha1, MPK6- mitogen-activated protein kinase6, RbohD/F- respiratory burst oxidase homolog D/F, PTEN2A- Phosphatase and TENsin homolog deleted, WER- Werewolf, GAPC- glyceraldehyde-3-phosphate dehydrogenase, RCN1 – roots curl in naphthylphthalamic acid 1, PARP1- poly(ADP-ribose) polymerase-1, GDSL-lipase- Gly-Asp-Ser-Leu lipase, PLAFP1- phloem lipid-associated family protein, PIG-P- phosphatidylinositol N-acetylglucosaminyltransferase subunit P, LHY- Late elongated hypocotyl, CCA1- circadian clock associated, AHL4- AT-hook motif-containing nuclear localized protein, LTP- long-term potentiation, ACBP2- acyl-CoA-binding protein, PCaP1,2- Plasma membrane cation binding protein, FAB1- Formation of aploid and binucleate cells, ACD11- Accelerated cell death, GLTP1- Glycosphingolipid transfer protein, PDLP5- plasmodesmata-located protein, ACBP2- acyl-CoA-binding protein.

## 4 Lipid-mediated tolerance of abiotic stresses

**Figure 7:**
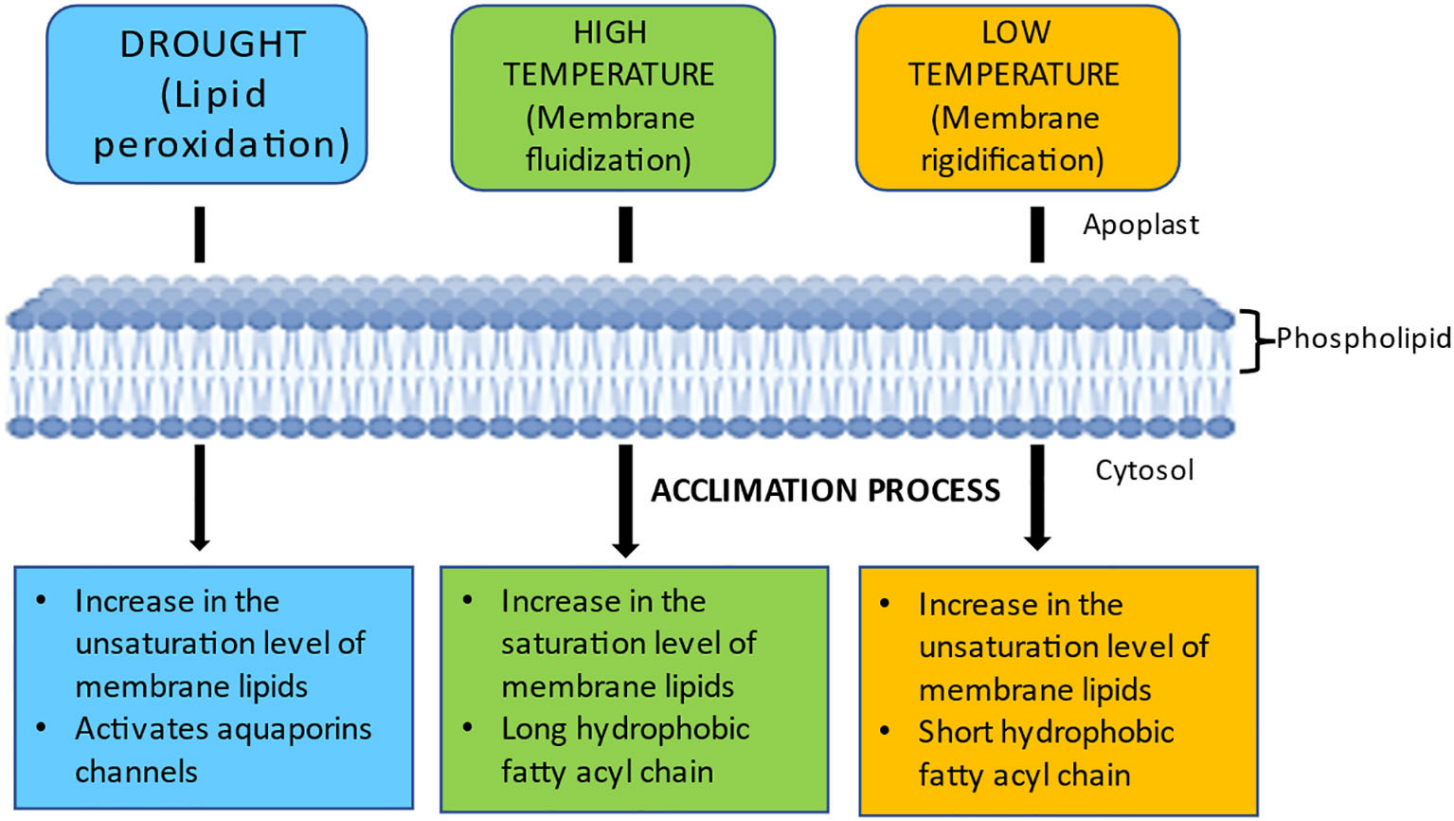
Variations in membrane fluidity in response to various abiotic stresses and acclimation processes. Drought affects the efficiency of water channels by increasing saturated fatty acyl chains and causing lipid peroxidation. Membrane rigidification occurs at low temperatures, while fluidization occurs at high temperatures. Plants acclimatize to drought by increasing their membrane lipid unsaturation levels to stabilize PM and activate their water channels (e.g., AQPs) to keep water moving through their cells. Plants adapt to high and low temperatures by decreasing and increasing membrane fluidity, respectively.

The degree of unsaturation of the lipid acyl chain (e.g., lipid packing) plays a crucial role in controlling membrane dynamics. Creating hydrogen connections between the membrane’s constituent parts inhibits membrane lipids from transitioning from a liquid crystalline state to a stiff gel state or vice versa (Wang et al., 2020). Unsaturated fatty acid chains bend at the cis-double bond, hindering membrane packing and resulting in membrane fluidization (He et al., 2018). The PM’s receptors and sensors are the primary sites for detecting and deciphering environmental stress signals. The response regulators transmit these signals downstream for appropriate molecular feedback, resulting in proper and adaptive responses to environmental stress (Barkla and Pantoja, 2010). Changes in the degree of fatty acid desaturation are crucial for plants to respond to different abiotic stressors. The principal polyunsaturated fatty acid species in membrane lipids are 16:3 and 18:3, with changes in those unsaturated fatty acid levels often dictating the membrane’s fluidity (Zhang et al., 2005; Wang et al., 2010). Maintaining high levels of fatty acid desaturation enables plants to stabilize membrane fluidity and reduce the damage inflicted on the membrane by abiotic stressors. **Figure 7** illustrates the changes in membrane lipid saturation due to various abiotic stresses and the associated acclimation processes.

In addition to their signaling functions, lipids also serve as stress mitigators, reducing the impact of abiotic stressors. For instance, membrane lipid modifications occur in response to stresses like cold, dehydration, and nutrient loss. This membrane remodeling helps maintain membrane characteristics impacting lipid dynamics, membrane integrity, and the activities of membrane-bound proteins (Samuels et al., 2008).

### 4.1 Lipid signaling in response to drought stress

Drought stress inhibits plant development by affecting various physiological and biochemical processes, including photosynthesis, chlorophyll production, nutrient metabolism, ion absorption and translocation, respiration, and carbohydrate metabolism (Jaleel et al., 2008; Li et al., 2011). Drought stress indicators include reduced leaf water potential and turgor pressure, stomatal closure, and reduced cell development and enlargement (Farooq et al., 2009). Lipid signaling molecules play a crucial role in controlling plant water demand, developing drought tolerance, and adapting to long-term drought. Dehydration leads to the production of PA, inositol phosphates, oxylipins, and sphingolipids (Bargmann et al., 2009b; Munnik and Testerink, 2009; Gasulla et al., 2013), highlighting the complexity of the stress response network and making it challenging to specify the particular physiological functions of different molecular lipid species. Controlling transpiration is a crucial defense mechanism against water scarcity, with most water loss occurring during transpiration through stomata. ABI1 is bound to the plasma membrane by PA binding, preventing it from moving to the nucleus, where it interacts with the transcription factor ATHB6, a regulator of the ABA response. The binding of PA and ABI1 suggests that ABA-mediated pathways require PA signaling (Zhang et al., 2004). The nitric oxide signaling cascade that causes stomatal closure also includes PA (Distéfano et al., 2008). Dehydrins, universally expressed in response to dehydration and connected with stress protection, are also activated by the upregulation of PA under drought stress (Koag et al., 2003). Both inositol phosphate 3 and inositol phosphate 6 affect stomata closure, while At5Ptase1, an inositol phosphatase activated in response to ABA, can block the IP3 pathway (Lemtiri-Chlieh et al., 2003). *Arabidopsis* fiery1 encodes another inositol phosphatase, which functions as a contraregulator of ABA (Xiong et al., 2001). Furthermore, the fiery1 mutation leads to the expression of ABA-responsive and stress-responsive genes, resulting in mutant plants with reduced resistance to cold, drought, and salt stress. Studies using transgenic plants have shown that IP3 levels play a crucial role in the expression of ABA-regulated genes, such as RD29A, KIN1, Cor15A, and HSP70, which were upregulated in plants with higher IP3 levels in response to stress treatments. Conversely, transgenic tomato lines with lower basal IP3 levels exhibited dramatically enhanced drought tolerance and changes to several metabolic and developmental alterations, such as higher net CO_2_ fixation and sucrose export to sink and storage tissues (Khodakovskaya et al., 2010). In addition to PA and IP3/IP6, sphingolipidsare also involved in controlling stomatal closure. ABA activates sphingosine kinases, which produce sphingosine-1-phosphate (S1P) that controls guard cell turgor. A downstream component of the S1P signaling pathway, heterotrimeric G-proteins, mediates ABA control of stomatal closure (Coursol et al., 2003). Upon binding to PA, phytosphingosine kinases (SPHKs) produce phytosphingosine phosphates, inducing stomatal closure in an ABA-dependent form (Guo et al., 2011; Guo et al., 2012). The link between oxylipin levels and drought tolerance was evidenced in *Arabidopsis* mutants, with lower oxylipin levels being more susceptible to drought (Grebner et al., 2013). In *Cannabis sativa*, there exists a partially shared biosynthetic pathway which is involved in the production of both oxylipins and cannabinoids. Drought stress has been found to have a significant impact on initiation of flowering in *Cannabis*. The production of cannabinoids, which are highly lipophilic molecules, changes under drought stress that led to decrease in the accumulation of cannabidol (CBD), while simultaneously increasing cannabigerol (CBG) levels (Park et al., 2022). Further research is necessary to fully understand the extent and mechanisms of this potential shared pathway and its implications for the production of oxylipins and cannabinoids. The involvement of several plant PLCs in abiotic stress signaling is strongly supported by functional analyses of these proteins using reverse genetic methods. For instance, increased drought endurance was evident in plants with *Zea mays* (maize) ZmPLC1, *Brassica napus* (rapeseed) BnPLC2, and *Nicotiana tabacum* (tobacco) PLC overexpression (Tripathy et al., 2012). Recent research found that constitutive AtPLC3 and AtPLC5 expression reduced stomatal aperture, preventing excessive water loss and enabling drought adaptation in *Arabidopsis* (Zhang et al., 2018). Concomitantly higher amounts of IP3 and PI(4,5)P2 were found in plant cells under osmotic stress, pointing to the simultaneous activity of PI-PLC and phosphoinositide kinase (Pokotylo et al., 2014). As exemplified by the above examples, lipid signaling molecules alter the activity and translocation of target proteins and, in some cases, cause conformational changes or the phosphorylation of downstream targets, altering their susceptibility to dehydration stress.

Research has shown that many plant species exhibit a response to drought stress by increasing the deposition of cuticular wax (Kosma and Jenks, 2007). The cuticle consists of two significant hydrophobic components: cutin and waxes (Kolattukudy, 1980). The waxes consist of long-chain aliphatic lipids, triterpenoids, and other minor secondary metabolites such as sterols and flavonoids. The composition of cuticular wax is believed to influence the barrier properties of cuticular transpiration. Within the cuticular wax biosynthetic pathways, C16 and C18 fatty acids are initially synthesized in the plastids and then transported to the cytoplasm. In the cytoplasm, they undergo further elongation to form very-long-chain fatty acids ranging from C20 to C34 in the endoplasmic reticulum. This elongation process is facilitated by a series of enzymes, including 3-ketoacyl-CoA synthetases (KCS), 3-ketoacyl-CoA reductases (KCR), 3-hydroxyacyl-CoA dehydratases, and trans-2-enoyl-CoA reductases (ECR) (Kunst and Samuels, 2009).The presence or absence of MYB96 in plant systems has been found to affect the content of cuticular wax, indicating that MYB96 integrates various signals related to drought stress and regulates the biosynthesis of cuticular wax. The myb96-1D mutant shows resistance to drought, whereas the myb96-1 mutant, deficient in MYB96, is highly susceptible to drought. Notably, the myb96-1D mutant exhibits a shiny surface on its leaves and stems, indicating the accumulation of cuticular waxes to a high degree. The MYB96 transcription factor directly binds to the promoters of genes that encode fatty acid elongation enzymes (Seo et al., 2009). The upregulation of genes involved in cuticular wax biosynthesis in the myb96-1D mutant suggests that MYB96 acts as a regulator of this process. Therefore, while cuticular wax biosynthesis may not be the primary mechanism for drought response, it likely becomes more important and potentially protective during severe drought stress.

The lipid matrix found in photosynthetic membranes contains 70–80% of MGDG and DGDG and it is crucial to regulate these lipids to ensure the continuous functioning of photosynthesis. The amount of DGDG and/or the DGDG to MGDG ratio are also vital in various essential cellular processes occurring within the chloroplast. These include protein folding, insertion of proteins, and intracellular protein trafficking (Dormann and Benning, 2002). A detailed analysis of plants with various susceptibilities to drought stress revealed that a decrease in MGDG levels was accompanied by an increase in oligogalactolipids, PI, and PA. These lipid profile modifications after desiccation were more evident in desiccation-tolerant species than in desiccation-sensitive ones (Gasulla et al., 2013). The interaction of DGDG and PG with external proteins stabilizes the PSII manganese cluster (Fujii et al., 2014). Changes in the DGDG/MGDG ratio may affect chloroplast membrane stability (Liu et al., 2017), as MGDG can be especially sensitive to drought stress (Kalisch et al., 2016). The MGDG synthetic gene knockout *Arabidopsis* mutant, mgd1, exhibited decreased MGDG expression but no change in PSII activity (Hernandez et al., 2016). However, in another study, the electrical conductivity of the mgd1 mutant’s thylakoid membrane increased, decreasing its photoprotective action (Cao et al., 2015). The growth and photosynthetic efficiency of an *Arabidopsis* mutant known as dgd1 were found to be adversely affected. This mutant exhibits a significant decrease in DGDG content (Dormann et al., 1995). Spring wheat subjected to drought stress had decreased PG content and increased DGDG/MGDG ratio. The author speculated that PC or PC-derived lipids might be delivered directly or indirectly to galactolipid biosynthetic plastids or that DAG may be phosphorylated into PA to produce DGDG. DGDG confers thermotolerance to plants due to its ability to stabilize bilayers, as evidenced by the inability of DGDG-deficient dgd1 mutant plants to adapt to high growth temperatures (Kobayashi, 2016). While the total lipid content of desiccation-tolerant plants does not vary, the membrane lipid composition does, and the amount of MGDG decreases. The production of phospholipids by DAG is one way to reduce MGDG levels. Another method involves converting MGDG to the DGD1/DGD2 pathway, followed by the production of oligogalactolipids from SFR2. This pathway significantly increases the stability of the chloroplast membrane by lowering the MGDG/DGDG ratio, which also helps maintain the bimolecular shape of lipids in membranes (Nakajima et al., 2018). Recent studies have focused on the modification and turnover of photosynthetic membrane lipids as a successful coping mechanism for adapting to and developing tolerance to adverse environments (Li et al., 2020; Yang et al., 2020). During periods of stress, plants decrease the amounts of MGDG, DGDG, PG, and total lipids, while the fatty acid composition of total lipids varied depending on the stress (Higashi et al., 2015). Plants frequently reduce tissue MGDG levels in response to drought, salt, cold temperatures, and aluminum stressors, with smaller declines in DGDG levels (Wang et al., 2014). Dehydration can decrease the amount of fatty acid desaturation in plants (Dakhma et al., 1995). During drought stress, a drought-tolerant maize cultivar maintained a higher level of fatty acid desaturation than a sensitive cultivar (Chen et al., 2018), which can provide plants with the capacity to stabilize membrane fluidity and reduce the damage inflicted by abiotic stressors on the membrane.

### 4.2 Lipid signaling in response to cold stress

Cold stress can cause membrane integrity loss and cellular dehydration, resulting in various phenotypic symptoms that impede plant reproduction and survival, including diminished leaf growth, leaf wilting, and leaf yellowing (Chinnusamy et al., 2007). Cell membranes are the primary site of cold-induced damage in plants (Kratsch and Wise, 2000). Low-temperature stress enhanced the relative conductivity of maize seedlings (Nguyen et al., 2009). Similarly, low-temperature stress increased the relative conductivity of tea leaves (Li et al., 2012). Altering the saturation rate of a specific lipid class by genetic modifications confirmed the clear relationship between membrane composition and chilling sensitivity (Nishida and Murata, 1996). Studies have shown that oligogalactolipids can minimize the effects of freezing stress.The SFR2 (SENSITIVE TO FREEZING 2) protein plays a vital role in preserving the integrity of chloroplast membranes when they are subjected to freezing temperatures (Roston et al., 2014). *SFR2*,a galactosyltransferase that transfers galactose moieties from MGDG molecules to other galactolipids, produces oligogalactolipids and DAG, which is then converted to triacylglycerol in the chloroplast envelope. This process of remodeling the lipid composition of chloroplast membrane in response to freezing assists in adapting the envelope membranes by elimination of excess polar lipids (Moellering et al., 2010).The *sfr2* mutant of *Arabidopsis* was extremely sensitive to freezing, leading to chloroplast rupture (Thorlby et al., 2004). Cold stress-induced membrane rigidification activates Ca^2+^ channels in the plasma membrane, with the extracellular Ca^2+^ influx increasing PA produced by PLC/DGK (phospholipase C/diacylglycerol kinase). Cold stress induces the synthesis of PA through the phospholipase D (PLD) and PLC/DGK pathways (Li et al., 2009). The PLC/DGK pathway mainly generates 16:0/18:2 and 16:0/18:3 PA species, while the PLD pathway generates 18:3/18:2 and 18:2/18:2 PA species (Vergnolle et al., 2005). Cold-tolerant plants can acclimate to low temperatures by increasing the FA unsaturation of PG molecules in the lipids of thylakoid membranes. Low-temperature stress (5°C) increased the PE and PA contents of *Z. mays* roots, indicating that *Z. mays* modifies its membrane lipid metabolism in response to temperature stress (Zhao et al., 2021). Barley cultivated at normal (25°C) and low (4°C) temperatures differed in glycerol lipid composition, with lower phospholipid and galactolipid contents in the cold-grown plants. Barley plants may be able to increase their cold resistance by modifying glycerol lipid content and FA saturation level to maintain the bilayer membrane structure and ensure membrane fluidity (Ruelland et al., 2015). Cold stress tolerance may also be influenced by the saturation level of membrane lipids (Routaboul et al., 2000). Studies on several species, including rice, maize, and tobacco, have also confirmed the relationship between membrane lipid saturation and cold tolerance (Kodama et al., 1995, Kodama et al., 1997; Murata and Wada, 1995). Peanut may increase cold tolerance by increasing unsaturated fatty acid levels and decreasing the buildup of saturated fatty acids (Liu et al., 2017). During cold stress, peanut plants significantly increased the concentrations of three unsaturated FAs—oleic acid (18:1), linoleic acid (18:2), and linolenic acid (18:3)—while those of saturated FAs, such as stearic acid (18:0) and palmitic acid (16:0), decreased. Four regulatory genes involved in lipid metabolism (AhACP1, AhmtACP3, AhACP4, and AhACP5) are closely associated with cold tolerance in peanut (Zhang et al., 2019). Linolenic acid and palmitic acid were closely associated with cold tolerance in winter wheat research (Dong-Wei et al., 2013). After cold acclimation, the ratio of unsaturated to saturated fatty acids increases. Nevertheless, evidence suggests that sphingolipid and oxylipin signaling are involved in low-temperature responses (Lynch, 2012; Hu et al., 2013). In *Arabidopsis*, a decrease in temperature from 22 to 4°C led to a rapid increase in phosphorylated long-chain phytosphingosine phosphate (PHS-P), which then activated MPK6 kinase, suggesting its involvement in an early response signaling pathway (Dutilleul et al., 2012). Jasmonate regulates the inducer of CBF expression C-repeat binding factor/DRE binding factor1 (ICECBF/DREB1) transcriptional pathway, indicating that jasmonate is an upstream signal that positively regulates *Arabidopsis* freezing tolerance (Hu et al., 2013). After 20 days of treatment at 4°C, the thylakoid membrane of sweet pepper expanded and distorted, the thylakoid grains split, and the starch grains grew (Kong et al., 2018). Studies have shown that low temperatures promote chloroplast deterioration, while chloroplast swelling improves cell matrix permeability (Moellering et al., 2010; Yang et al., 2011).

### 4.3 Lipid signaling in response to heat stress

Plants are constantly exposed to daily and seasonal temperature variations, necessitating the development of sophisticated mechanisms to detect and respond to environmental changes. High temperatures can destabilize proteins, enzymes, nucleic acids, biomembranes, and cytoskeletal structures (Asthir, 2015). A common effect of heat stress is tissue senescence, characterized by membrane damage caused by increased membrane lipid fluidity, lipid peroxidation, and protein degradation in various metabolic pathways (Savchenko et al., 2002). Plants have developed short-term stress avoidance and acclimation mechanisms and long-term phenological and morphological evolutionary adaptations to survive under high-temperature conditions, including shifting leaf orientation, transpiration cooling, or modifying membrane lipid composition (Wang et al., 2004). Membrane lipid saturation is considered a critical factor in determining a plant’s ability to tolerate high temperatures. The proportion of saturated fatty acids in membrane lipids affects the lipid melting point, regulating membrane fluidity and heat-induced membrane-fluidity changes. Therefore, plants increase their saturated and monounsaturated fatty acid concentrations in response to rising temperatures, regulating their metabolism and retaining membrane fluidity (Zhang et al., 2005). Heat stress alters the composition of membrane lipids, increasing lipid saturation levels in cell membranes (Schroda et al., 2015) to preserve membrane stability and improve heat tolerance (Larkindale and Huang, 2004). High-temperature stress increases membrane fluidity (Quinn, 1988). Temperature fluctuations significantly influence membrane-localized protein activity and membrane permeability to water, solutes, and proteins (Whiting et al., 2000). To counter the drop in phase transition temperature caused by unsaturated lipids, plants regulate the saturation level of membrane glycerolipids (Nishida and Murata, 1996). Plants can detect changes in ambient temperature using a complex network of sensors located in various cellular compartments. The resulting increase in membrane fluidity triggers lipid-based signaling cascades, Ca^2+^ influx, and cytoskeletal rearrangement, generating osmolytes and antioxidants. The *Arabidopsis* cyclic nucleotide-gated calcium channel (CNGC2) gene encodes a part of the membrane-bound cyclic nucleotide-gated Ca^2+^ channels, acting as the main thermosensors in terrestrial plant cells. The activation of these channels in the plasma membrane in response to an increase in the surrounding temperature triggers an ideal heat shock response (Saidi et al., 2009). Thylakoid lipids were observed to have a higher frequency of 16:0-acyl chains and lower frequency of 16:3-acyl chains under heat stress (Falcone et al., 2004). A shift toward higher saturation levels in membrane lipids has been observed during long-term heat exposure; however, short-term heat treatment appears insufficient for lipid remodeling (Balogi et al., 2005). The response of glycerolipids to moderate heat stress has been extensively studied. During this, plants exhibit an increase in galactolipids containing 18:2 (linoleate) and a decrease in galactolipids containing 18:3 (α-linoleate). In the endoplasmic reticulum and plasma membrane, there are increases in phospholipids containing 16:0 (palmitate), 18:0 (stearate), and 18:1 (oleate). Additionally, under moderate heat stress, there is an increase in lipid droplets containing triacylglycerol with 18:3 and 16:3 (hexadecatrienoic acid) (Falcone et al., 2004; Chen et al., 2006; Li et al., 2015; Higashi and Saito, 2019).

Moreover, heat stress activates MAPKs that regulate the expression of HSP genes (Horvath et al., 2012). High-temperature tolerance in *Arabidopsis* was correlated with a high DGDG/MGDG ratio (Chen et al., 2006). In maize, the drought-tolerant cultivar had a higher DGDG/MGDG ratio than the drought-sensitive cultivar, possibly related to delayed drought-induced leaf senescence (Chen et al., 2018). Similarly, drought stress increased the DGDG/MGDG ratio in *Arabidopsis* (Gigon et al., 2004). It is well-known that MGDG and DGDG are easily convertible, and the DGDG/MGDG ratio is correlated with plant growth stage. Thus, it is believed that while the DGDG/MGDG ratio may not determine a plant’s capacity to withstand stress, plants may adjust this ratio to some extent to enhance membrane stability under stress. In *Arabidopsis*, the process of heat acclimation can enhance the ability of plants to withstand extreme temperatures. In the case of heat acclimation, where plants are exposed to a temperature of 38°C for 2 hours, there is a reduction in the levels of MGDG and PG.

This decrease in MGDG content plays a role in maintaining the membrane’s integrity during heat shock (Deme et al., 2014). It is possible that the reduced MGDG is converted into triacylglycerols, which accumulate as a result of the heat acclimation process (Mueller et al., 2015).Changes in the degree of fatty acid desaturation are crucial for plants to respond to different abiotic stressors. The principal polyunsaturated fatty acid species in membrane lipids are 16:3 and 18:3, and their levels often dictate membrane fluidity (Zhang et al., 2005; Wang et al., 2010). Over time, heat stress increases the degree of membrane lipid saturation, while MGDG and DGDG are enriched in polyunsaturated acids (Higashi et al., 2018). Chinese cabbage Wucai (*Brassica campestris* L.) subjected to high temperatures had damaged chloroplast envelopes, expanded thylakoids, loosely organized grana lamellae, and larger and more chloroplast osmiophilic particles (Zou et al., 2017). Lipid osmiophilic particles also called plastoglobules (PGs) were found within chloroplasts, are closely associated with the photosynthetically active thylakoid membranes. They exhibit dynamic changes in size, number, and composition, indicating their active involvement in cellular processes. Their dynamic nature highlights their importance in the functioning and adaptation of chloroplasts to various physiological and environmental conditions (Austin et al., 2006). The important role of PGs has been suggested in enabling crucial adaptive responses of the thylakoid membrane to environmental changes by modifying the lipid composition of the thylakoid membrane, capturing and isolating catabolic intermediates, reducing the harmful effects of reactive oxygen species and generating stress signals (Rottet et al., 2015; Pralon et al., 2020).

## 5 Conclusion and future perspectives

**Figure 8:**
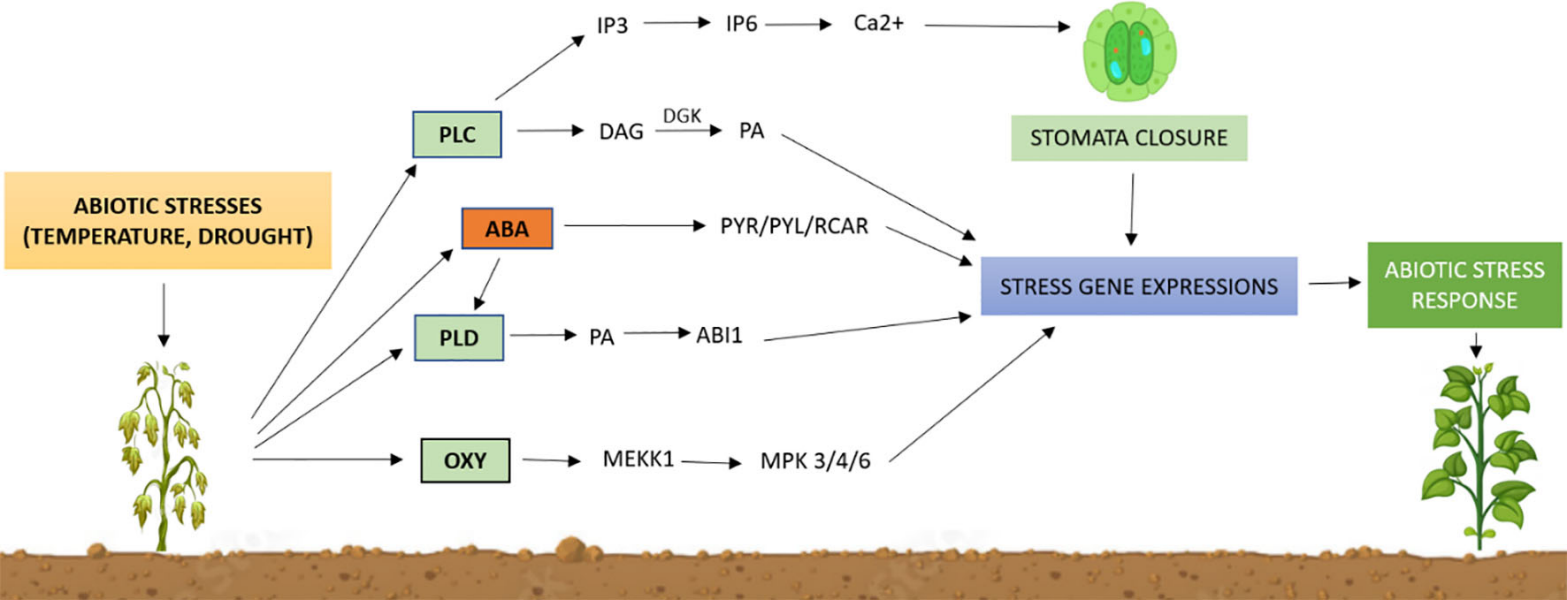
Outline of lipid-mediated pathways for abiotic stress tolerance in plants. Abiotic stresses activate membrane-bound phospholipases to generate lipid signaling molecules, such as phosphatidic acid (PA), inositol phosphates (IP3, IP6), and oxylipins, and induce the abscisic acid (ABA) signaling pathway. These lipids control protein activity, location, and structure while activating specific stress genes. Although the downstream mechanisms of lipid signaling remain poorly understood, changes in gene expression can cause physiological changes that lead to an environmental stress response. PLC, phospholipase C; PLD, phospholipase D; IP3, inositol triphosphate; IP6, inositol hexakisphosphate; DAG, diacylglycerol; DGK, diacylglycerol kinase; PA, phosphatidic Acid; PYR/PYL/RCAR, PYR/PYL/RCAR proteins that interact with ABI; MEKK1, MAPK kinase 1; MPK3/4/6, mitogen-activated protein kinase 3/4/6.

Lipid signaling molecules play crucial roles in plant responses to various abiotic stresses. **Figure 8** details the pathway of lipid-mediated abiotic stress tolerance. Lipid signaling molecules comprise many different types of signal transmitters. While PA and PIs have been extensively studied, other lipids such as sphingolipids, lysophospholipids, oxylipins, and NAEs are less understood but have recently garnered attention. However, identifying lipid signaling pathways in plants is challenging due to multifunctional enzymes, parallel isozyme networks, and a complex regulatory network involving several lipid species in response to stress. Advanced analytical detection techniques are needed to overcome these challenges and to identify direct downstream targets of lipid mediators.

Profiling minor signaling lipids and determining their interactions with downstream proteins *in vivo* are critical to understanding the function of lipid signaling in response to environmental stress. Synthesizing lipid signaling molecules into existing signaling stress pathways will enable a better interpretation of how plants respond to environmental stress, with the potential to create plants adaptable to harsh environmental conditions. Further studies on the role of lipid signaling in plant reactions to abiotic stress will advance fundamental plant science and crop breeding, helping to mitigate the impact of challenging environmental conditions. Additional research is need to characterize the changes in total lipid profiles in response to diverse abiotic and biotic stress conditions.

## Author contributions

The study was conceptualized by NL, PS, YA and AZ. PS, AG and AZ wrote the original draft. KS critically analyzed the manuscript and edited it for language. All authors contributed to the article and approved the submitted version.
